# Intriguing findings of liver fibrosis following COVID-19

**DOI:** 10.1186/s12876-021-01939-7

**Published:** 2021-10-11

**Authors:** Oksana Kolesova, Ieva Vanaga, Sniedze Laivacuma, Aleksejs Derovs, Aleksandrs Kolesovs, Maija Radzina, Ardis Platkajis, Jelena Eglite, Elvira Hagina, Seda Arutjunana, Davis Simanis Putrins, Jelena Storozenko, Baiba Rozentale, Ludmila Viksna

**Affiliations:** 1 Departments of Infectology, Rīga Stradiņš University, Riga, Latvia; 2 Institute of Microbiology and Virology, Joint Laboratory of Immunology and Immunogenetics, Rīga Stradiņš University, 5 Ratsupites Street, Riga, 1067 Latvia; 3 Riga East Clinical University Hospital, Riga, Latvia; 4 Faculty of Education, Psychology, and Art, University of Latvia, Riga, Latvia; 5 Faculty of Medicine, University of Latvia, Riga, Latvia; 6 Radiology Research Laboratory, Rīga Stradiņš University, Riga, Latvia; 7 Diagnostic Radiology Institute, Paula Stradina Clinical University Hospital, Riga, Latvia; 8 Department of Radiology, Rīga Stradiņš University, Riga, Latvia; 9 Central Laboratory Ltd., Riga, Latvia; 10 Department of Public Health and Epidemiology, Rīga Stradiņš University, Riga, Latvia

**Keywords:** Serological biomarker, COVID-19, Hyaluronic acid, Liver fibrosis index, Consequences

## Abstract

**Background:**

Studies on a new coronavirus disease (COVID-19) show the elevation of liver enzymes and liver fibrosis index (FIB-4) independently on pre-existing liver diseases. It points to increased liver fibrogenesis during acute COVID-19 with possible long-term consequences. This study aimed to assess liver fibrosis in COVID-19 patients by serum hyaluronic acid (HA) and FIB-4.

**Methods:**

The study included the acute COVID-19 group (66 patients, 50% females, mean age 58.3 ± 14.6), the post-COVID group (58 patients in 3–6 months after the recovery, 47% females, mean age 41.2 ± 13.4), and a control group (17 people, 47% females, mean age 42.8 ± 11.0). Ultrasound elastography was performed in the post-COVID and control groups.

**Results:**

Sixty-five percent of the acute COVID-19 group had increased FIB-4 (> 1.45), and 38% of patients had FIB-4 ≥ 3.25. After matching by demographics, 52% of acute COVID-19 and 5% of the post-COVID group had FIB-4 > 1.45, and 29% and 2% of patients had FIB-4 ≥ 3.25, respectively. Increased serum HA (≥ 75 ng/ml) was observed in 54% of the acute COVID-19 and 15% of the post-COVID group. In the acute COVID-19 group, HA positively correlated with FIB-4, AST, ALT, LDH, IL-6, and ferritin and negatively with blood oxygen saturation. In the post-COVID group, HA did not correlate with FIB-4, but it was positively associated with higher liver stiffness and ALT.

**Conclusion:**

More than half of acute COVID-19 patients had increased serum HA and FIB-4 related to liver function tests, inflammatory markers, and blood oxygen saturation. It provides evidence for the induction of liver fibrosis by multiple factors during acute COVID-19. Findings also indicate possible liver fibrosis in about 5% of the post-COVID group.

## Introduction

The studies on a new coronavirus disease (COVID-19) frequently show impaired liver function with elevated transaminases. In some cases, the elevation of liver enzymes was more than three times and reflected a liver injury independently on pre-existing liver diseases [1]. Autopsies of COVID-19 patients with biochemical evidence of hepatitis revealed macrovesicular steatosis, lobular necroinflammation, and mild portal inflammation in most cases [2]. Possible hepatotropism of the SARS-CoV-2 virus, systemic inflammatory response, hypoxic ischemia–reperfusion injury, and direct drug effect are the main reasons for impaired liver function and liver injury during COVID-19 [3, 4].

A liver biopsy can be very informative for the liver investigation at the time of the acute COVID-19 injury [5]. At the same time, obtaining liver tissue during active respiratory illness is technically and clinically challenging [4], especially in the view of the possibility of COVID-19 related coagulopathy [6, 7]. This most likely is a reason for only three biopsies performed in patients with COVID-19 presented in publications [8, 9]. Using non-invasive tests in assessing processes in liver in the early stage of COVID-19 can extend our knowledge in the pathogenesis of SARS-CoV-2 infection and provide the basis for applying these markers for monitoring acute illness.

Liver fibrosis index (FIB-4) is one of these tests, which is widely used in detecting a stage of fibrosis and monitoring of chronic liver diseases, including chronic viral hepatitis, HIV/HCV co-infection, and metabolic-associated fatty liver disease [10, 11]. Recent studies in patients with COVID-19 [12–14] showed that more than half of patients at admission to the hospital had increased FIB-4. The level of FIB-4 is thought to be related to SARS-CoV-2 viral load and levels of interleukin 6 (IL-6) and interferon gamma-induced protein (IP-10) [15]. These studies underline that FIB-4 at the early stage of COVID-19 disease is a predictor of a negative outcome and the need for mechanical ventilation in middle-aged patients. However, some authors [12, 13] point that FIB-4 may not reflect liver fibrosis due to transient elevation of transaminases during acute COVID-19. Simultaneously, these studies did not assess an association of FIB-4 with other parameters of liver fibrosis as serum hyaluronic acid (HA) or liver stiffness, measured by ultrasound elastography. The analysis of these relationships can reveal possible liver fibrosis and its mechanisms in acute COVID-19.

HA is one of the oldest serum biomarkers of liver fibrosis, which was revealed by Meyer and Palme [16]. Produced by fibroblasts and specialized connective tissue cells, HA is a mucopolysaccharide forming an extracellular matrix. In a healthy human, serum HA ranges from 0 to 75 ng/ml [17]. Hepatic stellate cells (HSC) are responsible for HA synthesis in the liver. Pro-inflammatory cytokines transform HSC into myofibroblasts, producing more HA than non-activated HSC [18]. The association between increased serum HA and a stage of liver fibrosis, described in chronic viral hepatitis B or C and HIV/HCV co-infection [11, 19–21], showed that serum HA is a useful marker of fibrogenesis. The enhanced production of HA in response to various viral, toxic, and inflammatory stimuli can reflect fibrotic processes in other tissue (for example, in the lungs). A previous study on COVID-19 [22] detected serum HA in association with the lung damage. However, currently there are no data on the association of HA with hepatic impairment in COVID-19 patients.

The assessment of the health status of people after COVID-19 is the additional issue of our study. COVID-19-associated hepatocellular and cholangiocellular injury may lead to activation of HSC and induction of fibrosis, which can cause long-term consequences [1].

Our study aimed to assess liver fibrosis in COVID-19 patients by serum HA and FIB-4 and to detect their relationship with liver function tests, inflammatory markers, and blood oxygen saturation. Possible liver fibrosis was assessed upon admission to the hospital and 3–6 months after the recovery (post-COVID). Additionally, in the post-COVID group, the measurements of HA and FIB-4 were combined with ultrasound elastography findings. The latter reflects the physical properties of the liver and is a validated non-invasive method for detecting and staging liver fibrosis [23, 24]. Unfortunately, the assessment of liver stiffness in the acute COVID-19 was not possible due to safety protocol limitations.

## Material and methods

### Participants

The study was conducted from September to December 2020. The Central Medical Ethics Committee, Riga, Latvia (protocol No. 01-29.1/2429), and the Ethics Committee of Rīga Stradiņš University, Latvia (protocol No. 6-1/07/14) provided approval for the study. All participants signed the informed consent form. The study was performed in accordance with the Declaration of Helsinki.

The cross-sectional, single-center study included 141 participants, divided into three groups: 66 patients with acute COVID-19 (acute COVID-19 group), 58 patients 3–6 months after acute COVID-19 (post-COVID group), and 17 participants without COVID-19 (control group). All patients with acute COVID-19 were treated in Riga East Clinical university hospital.

The diagnosis of SARS-CoV-2 infection was confirmed by positive real-time reverse-transcription polymerase chain reaction (RT-PCR) for SARS-CoV-2 nucleic acids in a nasopharyngeal swab. All participants in the control group had negative SARS-CoV-2 nucleic acids and negative anti-SARS-CoV-2 antibodies tests at the time of health status assessment. Data on the presence of comorbidities were obtained from medical documentation and by a survey.

### Clinical tests

The clinical tests included: white cells count (WBC), erythrocytes (RBC), platelet count (PLT), absolute CD4^+^ T lymphocyte count, erythrocyte sedimentation rate (ESR), C-reactive protein (CRP), ferritin, interleukin 6 (IL-6), alanine aminotransferase (ALT), aspartate aminotransferase (AST), lactate dehydrogenase (LDH), and creatinine. In acute patients, blood samples were received within 24 h of admission to the hospital. Data of blood oxygen saturation (SpO_2_) at admission to the hospital and the minimal level of SpO_2_ during hospitalization also were analyzed.

### Hyaluronic acid

Venous blood samples were centrifuged at 4000×*g* for 10 min, and serum was stored at − 80 °C until analysis. For acute patients, serum samples were obtained within 24 h after admission to the hospital. For all participants, serum samples were obtained on an empty stomach. Hyaluronic acid (HA) was detected by an enzyme-linked immunosorbent assay kit (Hyaluronan Quantikine ELISA Kit, R&D Systems, USA) following manufacturer instruction.

### Index of liver fibrosis (FIB-4)

To assess the level of liver fibrosis, FIB-4 was calculated [10]:

$${\text{FIB-4}} = \{ {\text{Age}}\left( {{\text{years}}} \right) \times {\text{AST}}\,{\text{(U}}/{\text{l)}}\} /\{ {\text{PLT}}\,( \times 10^{3} /{\upmu }{\text{l}}) \times [{\text{ALT}}\,({\text{U}}/{\text{l}})]^{{1/2}} \} .$$

The levels of FIB-4 were categorized according to validated cut-off levels [10]: FIB-4 < 1.45 (negative predictive of advanced liver fibrosis), FIB-4 = 1.45–3.24 (significant liver fibrosis), and FIB-4 ≥ 3.25 (advanced liver fibrosis).

### Ultrasound elastography

The ultrasound examination included 2D-shear wave elastography (2D-SWE) for quantitative analysis of fibrosis because pathologically increased liver stiffness in kPa correlates to decreased tissue elasticity. In addition, shear wave dispersion pattern (SWD) in (m/s)/kHz was used as an indicator of tissue viscosity and suggestive of inflammation [25]. The quantitative evaluation of both parameters was performed in a selected homogenous area of the liver parenchyma with at least five consequent measurements taken to obtain median values. Measurements were considered reliable if the interquartile range/median ratio (IQR/M) was lower than 0.3. Due to safety and availability concerns, SWE and SWD measurements were not performed for patients in the acute COVID-19.

### Statistics

The biomarker levels were presented as median values and interquartile range. The Kruskal–Wallis test was applied for a comparison of independent groups. Multiple pairwise comparisons used the Dunn-Bonferroni significance correction. The Wilcoxon test was used for the assessment of dynamics within a group. The relationships between variables were assessed by the Spearman rank correlation coefficient. All computations were performed with IBM SPSS Statistics 22.0.

## Results

### Demographics and clinical characteristic of groups

The acute COVID-19 group included 66 patients (50% females) aged between 26 and 82 (mean age was 58.3 ± 14.6). Unilateral pneumonia was diagnosed in 18%, and bilateral pneumonia in 70% of patients. Other patients in the acute COVID-19 group (12%) did not have radiologically confirmed pneumonia. Blood oxygen saturation ≤ 93% was observed in 62% of patients during the hospitalization period. COVID-19 progression to the acute respiratory distress syndrome (ARDS) was observed in two patients. Ten percent of patients were treated in the Intensive Care Unit, and two patients (3%) died. Comorbidities were presented in 65% of the acute COVID-19 group: 50% of patients had hypertension, 9%—chronic heart failure, 17%—diabetes, 8%—asthma, and 2%—pulmonary fibrosis. Chronic liver disease was reported in 6% of patients (one patient had fatty liver disease while three patients had chronic viral hepatitis B or C).

The post-COVID group included 58 patients (47% females) aged between 20 and 66 (mean age was 42.1 ± 13.4). Hypertension was present in 6% of patients, asthma in 5%, and 3% of patients had chronic liver disease (one patient had fatty liver disease, and one patient had chronic viral hepatitis C).

The control group included 17 people (47% females) aged between 26 and 63 (mean age was 42.8 ± 11.0). Between comorbidities, hypertension was present in 6% and asthma in 12% of the control group. There were no participants with a previous history of chronic liver disease.

### Clinical indicators in acute COVID-19 patients

Table 1 presents the clinical tests of patients upon admission to the hospital in the acute COVID-19 group. The analysis revealed changes in liver function in acute patients before starting any specific COVID-19 therapy. Acute COVID-19 patients had abnormal transaminases at admission to the hospital, 29% and 38% for ALT and AST, respectively. FIB-4 at the level 1.46–3.24 was presented in 27% of acute patients, and FIB-4 ≥ 3.25 was in 38% of patients. The normal FIB-4 index (< 1.45) was identified in a third of patients. A half of patients (54%) had abnormal HA levels (cut-off for HA ≥ 75 ng/ml).

**Table 1:** Baseline clinical indicators in the acute COVID-19 group (n = 66)

Parameters	Median (IQR)	Missing data, n
Indicators of fibrosis		
FIB-4 (IQR)	2.51 (1.30; 3.56)	–
FIB-4 ≤ 1.45	35%	
FIB-4 1.46–3.24	27%	
FIB-4 ≥ 3.25	38%	
HA, ng/ml (IQR)	80.75 (45.94; 157.62)	–
HA ≥ 75 ng/ml	54%	
Enzymes		
ALT, U/l (IQR)	27 (18; 43)	–
ALT > 40 U/l	29%	
AST, U/l (IQR)	35 (22; 50)	–
AST > 40 U/l	38%	
LDH, U/l (IQR) (reference range 135–225)	288 (213; 416)	4
Blood cells		
WBC, × 10^3^/µl (IQR) (reference range 4.0–9.0)	5.12 (4.19; 6.84)	–
RBC, × 10^6^/µl (IQR) (reference range: males 4.0–5.5, females 3.9–5.0)	4.58 (4.22; 4.90)	–
Platelet, × 10^3^/µl (IQR) (reference range150–400)	185 (143; 229)	–
CD4^+^ T lymphocytes, cells/mm^3^ (IQR)	471 (312; 646)	16
CD4^+^ < 400 cells/mm^3^	42%	
Inflammatory markers		
CRP, mg/l (IQR) (reference range < 5.0)	41.82 (12.34; 86.98)	–
Ferritin, ng/ml (IQR) (reference range 30–400)	365.6 (171.3; 900.6)	1
IL-6, pg/ml (IQR) (reference range < 5.0)	16.10 (8.15; 44.50)	13
ESR, mm/h (IQR) (reference range < 20)	27.0 (18.0; 40.8)	2
Renal function		
Creatinine, µmol/l (IQR) (reference range: male 62–106, female 44–80)	75 (65; 93)	2
Oxygen saturation		
SpO_2_ at admission, %	95 (91; 98)	9
SpO_2_ ≤ 93% at admission	30%	
Lowest SpO_2_ during hospitalization	92 (88; 95)	3

IQR, interquartile range; HA, hyaluronic acid; WBC, leucocytes; RBC, erythrocytes; CRP, C-reactive protein; ALT, alanine aminotransferase; AST, aspartate aminotransferase; LDH, lactate dehydrogenase; IL-6, interleukin 6; ESR, erythrocytes sedimentation rate; SpO_2_, oxygen saturation in blood

In the acute COVID-19 group, the medians of LDH, CRP, ferritin, IL-6, and ESR at admission to the hospital were higher than the upper limit of a reference interval. Forty-two percent of acute patients at admission had CD4^+^ T lymphocyte count lower than 400 cells/mm^3^. A third of patients at admission had oxygen saturation ≤ 93%. The median levels of blood cells and creatinine in acute patients were in the reference interval.

The correlation analysis revealed relationships between markers of liver fibrosis, liver ferments, inflammatory markers, and oxygen saturation in the acute COVID-19 group (Table 2).

**Table 2:** Spearmen rank correlation coefficients in the acute COVID-19 group

	Age	FIB-4	HA	AST	ALT	LDH	CRP	IL-6	Ferritin
FIB-4 n	0.48*** 66	**–**							
HA n	0.51*** 66	0.50*** 66	**–**						
AST n	0.23 66	0.60*** 66	0.52*** 66	**–**					
ALT n	0.05 66	0.19 66	0.39** 62	0.71*** 66	**–**				
LDH n	0.36** 66	0.41*** 62	0.52*** 62	0.51*** 66	0.33** 62	**–**			
CRP n	0.34** 66	0.14 66	0.20 66	0.29* 66	0.11 66	0.53** 62	**–**		
IL-6 n	0.32* 53	0.41** 53	0.35** 53	0.48*** 53	0.30* 53	0.51*** 61	0.49*** 53	**–**	
Ferritin n	0.13 65	0.30* 65	0.31* 65	0.55*** 65	0.36** 65	0.53*** 61	0.45*** 57	0.42** 52	**–**
SpO_2_ n	− 0.35*** 57	− 0.23 57	− 0.49*** 57	− 0.36** 57	− 0.34** 57	− 0.67** 53	− 0.57*** 57	− 0.50*** 44	− 0.46*** 56

HA, hyaluronic acid; AST, aspartate aminotransferase; ALT, alanine aminotransferase; LDH, lactate dehydrogenase; CRP, C-reactive protein; IL-6, interleukin 6; SpO_2_, blood oxygen saturation. ***p < 0.001; **p < 0.01; *p < 0.05

In the acute COVID-19 group, HA correlated with all parameters except for CRP. HA was higher in patients with higher AST (r_s_ = 0.52, *p* < 0.001), ALT (r_s_ = 0.39, *p* = 0.001), FIB-4 (r_s_ = 0.50, *p* < 0.001), LDH (r_s_ = 0.52, *p* < 0.001), IL-6 (r_s_ = 0.35, *p* = 0.009), ferritin (r_s_ = 0.31, *p* = 0.012), and lower oxygen saturation at admission (r_s_ = − 0.49, *p* < 0.001).

FIB-4 correlated also with AST (r_s_ = 0.60, *p* < 0.001), LDH (r_s_ = 0.41, *p* < 0.001), ferritin (r_s_ = 0.30, *p* < 0.05), and IL-6 (r_s_ = 0.41, *p* < 0.01). Oxygen saturation at admission to the hospital negatively correlated with all parameters except for FIB-4.

### Differences between groups

Considering the revealed correlation between age and FIB-4 and HA level, we compared all the parameters in three groups matched by demographics. The age of acute patients was adjusted to other groups.

FIB-4, HA, AST, LDH, CRP, ferritin, IL-6, and ESR at admission to the hospitals (acute COVID-19 group) were higher than in the post-COVID and the control groups (Table 3). Adjusted estimates of the effect size (eta-square) for nonparametric comparisons [26] and an approximate estimate of the statistical power [27] for each marker indicated that effect sizes for FIB-4, LDH, CRP, IL-6, and ESR were no less than 0.14 or large [26] and provided sufficient statistical power (> 0.80). Effect sizes for AST and ferritin also were large, while the effect size for HA was medium. However, these three findings demonstrated lowered statistical power.

**Table 3:** Baseline clinical indicators in independent groups matched by demographics

Parameters	Patients group			Kruskal–Wallis, *p* value	η^2^
	COVID-19 (n = 31)	Post-COVID (n = 58)	Control (n = 17)		
Demographics					
Age, years ± SD (Min–max)	46.1 ± 11.0 26–65	41.2 ± 13.4 20–66	42.8 ± 11.0 26–63	2.82 *p* = 0.244	0.01
Females	48%	47%	47%		
Indicators of fibrosis					
FIB-4 (IQR)	1.45^a^ (0.85; 3.31)	0.59^b^ (0.45; 0.77)	0.62^b^ (0.54; 1.01)	30.17 *p* < 0.001	0.28
FIB-4 ≤ 1.45	52%	95%	94%		
FIB-4 1.46–3.24	19%	3%	6%		
FIB-4 ≥ 3.25	29%	2%	0%		
HA, ng/ml (IQR)	51.80^a^ (31.84; 81.50)	35.95^b^ (15.93; 70.18)	26.30^b^ (16.35; 34.80)	11.91 *p* = 0.003	0.10
HA ≥ 75 ng/ml	32%	15%	0%		
Enzymes					
ALT, U/l (IQR)	26 (14; 46)	26 (20; 36)	24 (20; 33)	3.58 *p* = 0.167	0.02
AST, U/l (IQR)	30^a^ (21; 42)	19^b^ (17; 24)	20^b^ (18; 28)	16.28 *p* < 0.001	0.14
LDH, U/l (IQR)	253.5^a^ (184.0; 374.8)	166.0^b^ (154.8; 190.5)	185.0^b^ (154.0; 212.5)	66.99 *p* < 0.001	0.25
Blood cells					
WBC, × 10^3^/µl (IQR)	5.10 (4.19; 6.99)	5.55 (4.73; 6.44)	6.48 (5.26; 7.52)	3.58 *p* = 0.167	0.02
RBC, × 10^6^/µl (IQR)	4.46^a^ (4.21; 5.02)	4.87^b^ (4.59; 5.15)	4.63^ab^ (4.30; 5.20)	7.01 *p* = 0.030	0.05
Platelet, × 10^3^/µl (IQR)	183^a^ (144; 212)	248^b^ (200; 271)	251^b^ (213; 294)	16.55 *p* < 0.001	0.14
CD4^+^ T lymphocytes, cells/mm^3^ (IQR)	479^a^ (401; 682)	830^b^ (665; 1035)	860^b^ (710; 1020)	20.14 *p* < 0.001	0.18
CD4^+^ < 400 cells/mm^3^	24%	5%	0%		
Inflammatory markers					
CRP, mg/l (IQR)	28.00^a^ (5.90; 68.80)	0.65^b^ (0.31; 1.77)	0.60^ab^ (0.31; 2.08)	49.00 *p* < 0.001	0.46
Ferritin, ng/ml (IQR)	291.7^a^ (142.8; 918.1)	108.5^b^ (72.8; 165.3)	108.0^b^ (47.9; 304.5)	16.79 *p* < 0.001	0.15
IL-6, pg/ml (IQR)	11.18^a^ (5.07; 28.08)	3.45^b^ (2.35; 4.90)	3.5^b^ (2.0; 4.3)	35.55 *p* < 0.001	0.33
ESR, mm/h (IQR)	23.0^a^ (18.0; 43.5)	2.0^b^ (2.0; 4.3)	2.0^b^ (2.0; 2.5)	66.99 *p* < 0.001	0.64
Renal function					
Creatinine, µmol/l (IQR)	76 (65; 87)	71 (65; 78)	74 (63; 85)	1.39 *p* = 0.498	0.01

Different superscripts indicate significant differences between subgroups. IQR, interquartile range; HA, hyaluronic acid; WBC, leucocytes; RBC, erythrocytes; CRP, C-reactive protein; ALT, alanine aminotransferase; AST, aspartate aminotransferase; LDH, lactate dehydrogenase; IL-6, interleukin 6; ESR, erythrocytes sedimentation rate; η^2^, estimate of the effect size for the Kruskal–Wallis test. Missing data reduced the number of measures in the COVID-19 group in LDH (n = 28), CD4^+^ (n = 21), IL-6 (n = 26), and ESR (n = 29)

Platelets and CD4^+^ T lymphocyte count were lower in the acute COVID-19 group than in the post-COVID and the control groups. Both comparisons revealed large effect sizes, but the statistical power was lowered for platelets and sufficient for CD4^+^ T lymphocyte count. Moreover, CD4^+^ T lymphocyte count < 400 cells/mm^3^ was observed in 24% of the COVID-19 group and 5% of the post-COVID group. ALT, WBC, and creatinine had no differences between groups. In the post-COVID group, the patients had higher erythrocyte count than in the control and acute COVID-19 groups.

Assessment of liver fibrosis showed that the patients with acute COVID-19 had a higher FIB-4 than in post-COVID and control groups. Intermediate liver fibrosis index (FIB-4 = 1.46–3.24) was observed in 19% of acute patients, in 3% of post-COVID, and 6% of the control group. At the same time, FIB-4 ≥ 3.25 was identified in 29% of acute and only in 2% of post-COVID patients. In the control group, a high level of liver fibrosis was not observed.

A similar tendency was observed in HA measurements. Patients with acute COVID-19 had a higher HA level than participants in post-COVID and the control groups. The level of HA ≥ 75 ng/ml was observed in 32% of acute COVID-19 patients, in 15% of the post-COVID group. In the control group, a high HA level was not observed.

It should be noted that two patients in the post-COVID group were with chronic liver diseases in the anamnesis. However, their FIB-4 levels (0.84 and 1.26) and HA (74.56 and 69.10 ng/ml) were not elevated.

### Dynamics in a small group

Ten patients of the post-COVID group presented with some blood parameters at two temporal points: at admission to the hospital and after 3–6 months after recovery (Table 4). It should be noted that a small group of patients provided a provisory assessment of change and estimates of the effect size.

**Table 4:** Dynamics of clinical tests in patients with COVID-19 at two-time points (n = 10)

Parameters	Acute COVID-19 (baseline)	Post-COVID	z	*p*	η^2^
WBC, × 10^3^/µl (IQR)	5.39 (4.21; 6.95)	4.86 (4.63; 6.70)	− 0.56	0.575	0.03
RBC, × 10^6^/µl (IQR)	4.47 (4.35; 4.74)	4.69 (4.57; 4.89)	− 2.30	0.022	0.53
PLT, × 10^3^/µl (IQR)	205 (162; 245)	260 (230; 285)	− 1.96	0.051	0.38
ESR, mm/h	19 (12; 43)	2 (2; 5)	− 2.37	0.018	0.56
ALT, U/l (IQR)	21 (12; 47)	25 (16; 35)	− 0.05	0.959	0.00
AST, U/l (IQR)	23 (20; 42)	19 (16; 26)	− 1.94	0.052	0.38
FIB-4 (IQR)	0.99 (0.74; 3.11)	0.65 (0.45; 0.89)	− 2.19	0.028	0.48
CRP, mg/l (IQR)	30.64 (3.30; 56.89)	1.92 (1.03; 5.44)	− 2.70	0.007	0.73
Creatinine, µmol/l (IQR)	79 (57; 91)	73 (63; 82)	− 0.42	0.678	0.02

IQR, interquartile range; WBC, leucocytes; RBC, erythrocytes; PLT, platelets; ESR, erythrocytes sedimentation rate; ALT, alanine aminotransferase; AST, aspartate aminotransferase; CRP, C-reactive protein; η^2^, estimate of the effect size for the Wilcoxon signed-rank test

The longitudinal investigation of these patients showed that inflammatory markers (ESR, CRP) and FIB-4 were higher in the acute COVID-19 than in the post-COVID period. Erythrocytes were higher in the post-COVID than in the acute COVID-19. Leucocyte count, ALT, and creatinine did not show differences between the two measurements. AST and platelets showed marginal differences (*p* = 0.052 and *p* = 0.051, respectively) with higher AST and lower PLT in acute COVID-19 than in the post-COVID period. Considering both parameters are included in the FIB-4 index, these marginal tendencies added to higher FIB-4 in acute COVID than after recovery.

### Ultrasound elastography for the post-COVID and control groups

There were no differences between the post-COVID and control groups in the measurements of SWE, z = − 0.08, *p* = 0.937, and SWD, z = − 0.72, *p* = 0.474. SWE was 4.60 kPa (IQR: 4.00; 5.53) in the post-COVID group and 4.55 kPa (IQR: 4.15; 4.88) in the control group. SWD was 11.75 (m/s)/kHz (IQR: 10.53; 12.75) and 12.10 (m/s)/kHz (IQR: 11.23; 13.20) in the post-COVID and the control groups, respectively. At the same time, SWE correlated with HA (r_s_ = 0.30, *p* = 0.021) and ALT (r_s_ = 0.32, *p* = 0.013), while the correlation with FIB-4 was not significant (r_s_ = 0.15, *p* = 0.26) in the post-COVID group. There were no significant correlations between elastography data and FIB-4 and HA in the control group.

## Discussion

Our findings indicated a high percentage of patients with impaired liver function and increased markers of liver fibrosis in acute COVID-19. Intercorrelations among most of the parameters under investigation pointed at the multifactorial liver injury. Longitudinal data in a small group showed liver function restoration after the recovery. However, about 5% of patients in the post-COVID group had signs of liver fibrosis independently of pre-existing liver diseases, and they should be monitored for a longer time.

### FIB-4 level at baseline and its association with other parameters

Using the cut-off levels of FIB-4 [10] revealed a high percentage of COVID-19 patients (65%) with abnormal liver fibrosis index (FIB-4 > 1.45). Interpretation of FIB-4 levels showed that 38% of patients had advanced liver fibrosis (FIB-4 ≥ 3.25) at admission, and 27% of patients had an intermediate level of FIB-4 (1.46–3.24). Analysis of differences between patients with acute COVID-19, after recovery, and the control group matched by demographics also showed a higher percentage of patients (52%) with FIB-4 > 1.45 in acute COVID-19 than in post-COVID groups (5%) and in the control group (6%). It is necessary to note that 29% of acute COVID-19 and 2% of post-COVID groups had dramatically increased FIB-4 (≥ 3.25). This FIB-4 level was not presented in the control group.

Similar findings in FIB-4 were described in Ibáñez-Samaniego et al. [12] and Sterling et al. [13] studies, revealing abnormal FIB-4 in 76% of acute patients at baseline [12] and FIB-4 > 3.25 in 42% of patients [13]. On the one hand, increased FIB-4 can reflect underdiagnosed pre-existing chronic liver disease [12, 13, 15]. In our study, pre-existing liver disease were reported only in 6% of acute COVID-19 patients, similarly to Sterling et al. [13]. On the other hand, FIB-4 in acute COVID-19 can reflect systemic inflammation because of positive correlations with IL-6 and IP-10 [15]. In our study, a higher FIB-4 was observed in acute patients with a higher level of AST, LDH, IL-6, and ferritin that is in line with Li et al. [15]. Moreover, we can conclude that FIB-4 reflects COVID-19 severity associated with hepatocellular damage (increased AST), systemic inflammation (increased IL-6 and ferritin), and lung damage (increased LDH), observed during acute SARS-CoV-2 infection [28].

The dynamics of FIB-4 in COVID-19 were also analyzed in previous studies [12, 15]. Li et al. [15] showed that, during hospitalization, FIB-4 peaked and then normalized in the survival group but failed to normalize in the death group. Ibáñez-Samaniego et al. [12] analyzed FIB-4 values in a small group of patients 6 months before the diagnosis of COVID-19 and upon admission to the hospital and observed that FIB-4 categories did not change between the time points. In our study, we have investigated the level of FIB-4 at admission to the hospital and in 3–6 months after the recovery. In the small group of patients, we have revealed a decrease of FIB-4 after discharging from the hospital. It concurs with Li et al. [15]. Moreover, these results confirm the relationship between FIB-4 and the level of inflammation.

### Serum HA and its association with other parameters

The analysis of serum HA revealed that half of the patients at admission to the hospital had HA level ≥ 75 ng/ml. It was higher in patients with higher FIB-4, AST, ALT, LDH, IL-6, and ferritin and lower oxygen saturation at admission that indicated the multifactorial determination of an increase of HA in serum, including hepatocellular damage, hypoxia, and inflammation. After matching by demographics, a higher HA level was also observed in the acute COVID-19 group than in the post-COVID group. Increased serum HA and its association with the course of COVID-19 and poor prognosis previously was shown in another study [22]. In addition to these findings, Ding et al. [22] described an increased level of procollagen type III and IV and laminin in patients with COVID-19, which may lead to the possible development of pulmonary fibrosis.

Increased serum HA during COVID-19 can be associated with the possible development of ARDS [29, 30]. The SARS-CoV-2 virus directly infects alveolar epithelial cells and induces the increase of pro-inflammatory cytokines in the lung tissue. These cytokines are responsible for the increased production of HA by fibroblasts, which lead to the accumulation of HA in alveolar space, development of hyaline membranes, and ARDS [30]. In our study, only two acute COVID-19 patients had ARDS. Therefore, we can conclude that revealed relationships between HA and FIB-4 and liver enzymes point to increased fibrogenesis in the liver.

Liver fibrosis is a wound-healing process in response to acute or chronic liver injury with increasing deposition of extracellular matrix and decreasing its degradation [18, 31]. Association between HA and FIB-4, observed in the acute COVID-19 group, and relationships between HA and the stage of liver fibrosis [19, 31, 32] lead to a conclusion that high HA and FIB-4 during COVID-19 can be related to increased liver fibrogenesis. Moreover, the level of liver fibrogenesis is associated with the level of hepatocellular damage, inflammation, and hypoxia, described as possible mechanisms of liver injury and fibrosis in the acute COVID-19 [1, 3].

### Assessing COVID-19 consequences

In the post-COVID group 5% of patients had increased FIB-4, and 2% of them had advanced liver fibrosis (FIB-4 ≥ 3.25) despite the normal range of inflammatory markers. Similarly, serum HA > 75 ng/ml was observed in 15% of post-COVID patients. The analysis also demonstrated that increased FIB-4 and HA levels in the post-COVID group were observed independently of pre-existing liver diseases in anamnesis.

Data of ultrasound elastography provided additional information regarding the physical properties of liver parenchyma in the post-COVID group. Based on previous data [23, 24], liver stiffness measurements reflect the presence of liver fibrosis. Therefore, the association between a higher level of HA and higher SWE values confirms the increased liver fibrogenesis in the post-COVID group.

Liver disease in the anamnesis of two patients from the post-COVID group demonstrated no visible effect on the level of HA, FIB-4, SWE, and SWD. Therefore, the observed elevation of FIB-4 in 5% of patients in that group can be associated with consequences of COVID-19 or previously underdiagnosed liver diseases. As a result, there is a need for further exploration of recovery after COVID-19. Simultaneously, a practical suggestion is a greater focus of the national healthcare system on early diagnostics and prevention.

### Limitations

The study has visible limitations. First, the sample size was limited by the number of recovered patients during the project. The temporal interval of 3–6 months was reached in a small number of patients because of a relatively favorable epidemiological situation in the country. The number of acute patients also is relatively small because of the absence of or fragmented data at admission to the hospital. An additional problem was limited information regarding pre-existing chronic liver diseases. Second, the size of the control group and the number of longitudinally investigated patients were also limited. Extending samples and involving new patients was limited by a strict time frame of the project and redirecting financial resources to coping with epidemiological threats and challenges.

## Conclusions

More than half of acute COVID-19 patients had increased serum HA and liver fibrosis index FIB-4 upon admission to the hospital independently to pre-existing chronic liver disease and development of ARDS. Moreover, in acute COVID-19 patients, serum HA is related to FIB-4, liver function tests, inflammatory markers, and blood oxygen saturation. It suggests impaired liver function and increased liver fibrogenesis in the acute COVID-19, determined by hypoxia and inflammation. In most patients, the liver injury is transitory. However, a follow-up (after 3–6 months) revealed signs of development of liver fibrosis in some individuals. Further studies should focus on factors predisposed to COVID-related consequences for liver functioning.

## Data Availability

Datasets of the current study are available from https://doi.org/10.48510/FK2/B4HPSJ.
